# Antiaging Properties of Exosomes from Adipose-Derived Mesenchymal Stem Cells in Photoaged Rat Skin

**DOI:** 10.1155/2020/6406395

**Published:** 2020-12-21

**Authors:** Jun-Xian Liang, Xuan Liao, Sheng-Hong Li, Xiao Jiang, Ze-Hua Li, Yin-Di Wu, Li-Ling Xiao, Guang-Hui Xie, Jian-Xing Song, Hong-Wei Liu

**Affiliations:** ^1^Department of Plastic Surgery, The First Affiliated Hospital of Jinan University, Innovative Technology Research Institute of Tissue Repair and Regeneration, Key Laboratory of Regenerative Medicine, Ministry of Education, Guangzhou Guangdong Province 510630, China; ^2^Department of Plastic Surgery, Changhai Hospital, The Second Military Medical University, Shanghai, China

## Abstract

Adipose-derived stem cells (ADSCs) have been documented as possible candidates for skin rejuvenation. However, the effects of ADSC-derived exosomes on photoaged skin remain to be fully elucidated. This study was aimed at determining the antiaging effects of ADSC-derived exosomes on photoaged skin. Human ADSCs were isolated from the adipose tissue of healthy women and cultured in vitro. Then, exosomes were extracted from the cultured ADSCs, purified by ultracentrifugation, and verified by examination of cell morphology using transmission electron microscopy and the identification of specific biomarkers. Meanwhile, the optimal exosome concentration and treatment time were selected. The photoaged skin model was created by subjecting Sprague-Dawley rats to ultraviolet B radiation. Exosomes were injected into the photoaged skin in a single therapeutic dose. The thickness of the epidermis and dermis was observed by HE staining. The relative mRNA expression of type I collagen, type III collagen, and matrix metalloproteinases (MMP-1 and MMP-3) was determined by real-time PCR. In the rat model of photoaged skin, the injected exosomes markedly decreased the epidermal thickness and increased the dermal thickness of the photoaged skin 7 days after treatment. Moreover, the proportion of the stratum corneum of the epidermis was decreased. Furthermore, real-time RT-PCR showed that the mRNA expression of type I collagen was increased and that of type III collagen, MMP-1, and MMP-3 was decreased. Our results demonstrate that ADSC-derived exosome treatment could significantly improve skin photodamage and that ADSC-derived exosomes may be a potential agent for photoaged skin treatment.

## 1. Introduction

Aging of the human body is inevitable with increasing age and is first observed more easily in the skin, which is the largest and most superficial organ in the human body. The reasons for this can be attributed to endogenous (gene inheritance and expression) as well as exogenous factors (various effects of skin exposure to the external environment) [1]. Under the combined effect of these two factors, the aging skin changes its appearance, function, and structure. Photoaging is an important exogenous factor, and UVB contained in sunlight plays an essential role in wrinkles caused by skin aging [1]. Photoaging is caused by light radiation and presents as leathery, rough wrinkles, irregular age spots, and hyperkeratosis. On histology, it presents as uneven thickening of the epidermis, disorganization of the capillary network of the skin, decreased type I collagen content in the skin, relative increase in type III collagen content, increased reticular fibers, denaturation of elastic fibers, and accumulation of sebaceous glands into masses. In addition, UV irradiation can also stimulate inflammatory reactions in the dermis and activate perivascular inflammatory mediators and cytokines, thereby producing lytic enzymes that slowly degrade dermal components. These changes are a process that occurs slowly over time with exposure to light (mainly ultraviolet light). During skin photoaging, the state of imbalance due to continuous or repeated proteolysis of the extracellular matrix causes incomplete repair of the dermis and is also an important cause of wrinkle formation [2].

In recent years, skin photoaging research and treatment have advanced, and the application of stem cells has become more and more extensive. With the development of stem cell transplantation for clinical wound treatment, some shortcomings in its clinical application have gradually emerged. For example, stem cells must be transplanted within a short period of time after extraction because of their demanding conditions for survival, making their use and preservation inconvenient. Stem cell transplantation from allogeneic or xenogeneic sources may provoke significant immune rejection and may increase the risk of viral infection to the host. In addition, related studies have found that direct differentiation and regeneration to promote wound healing are not the principal roles of stem cells in promoting wound healing. Instead, the primary mechanism through which stem cells promote the repair of damaged tissue is through the paracrine secretion of a number of active substances by the damaged tissue, of which exosomes are an important component. Exosomes have similar functions in wound healing and treatment as stem cell transplantation [3] and have an application potential due to long-term preservation, stable physiological properties, wide range of sources, and large-scale extraction. They are membranous bodies with diameters ranging from 30 to 150 nm. Stem cell-derived exosomes have similar biological functions to their parent stem cells [4] and contain a large number of genetic materials with their functional phases, and their contents can vary with the type of secreting cells. They contain mainly proteins, microRNAs, RNA, DNA, and lipids. However, few studies have reported the effects of exosomes on photoaging skin. In this study, we used subcutaneous injection of exosomes to treat skin photoaging in a UVB-induced skin photoaging model in Sprague-Dawley (SD) rats and observed the effect of exosomes on photoaging skin.

## 2. Materials and Methods

### 2.1. Sample Source

Adipose tissue was obtained from 6 young healthy women, aged 28–34 years, from the Department of Plastic Surgery, the First Affiliated Hospital of Jinan University, after obtaining informed consent from each patient.

### 2.2. *In Vitro* Culture of ADSCs from Adipose Tissue

Fat aspiration was conducted to extract subcutaneous fat tissue from the thigh or lower abdomen of healthy adult females. Fibrous connective tissue components were removed, and the tissue was placed in a centrifuge tube and washed 2–3 times with phosphate-buffered saline (PBS, Gibco Company, USA). An equal volume of 1% type I collagenase (Gibco Company, USA) was added, and digestion with agitation was allowed to proceed for 30 min at 37°C. The mixture was centrifuged at 300 × *g* for 10 min. Complete medium (Dulbecco's modified Eagle's medium (Gibco Company, USA) plus 10% fetal bovine serum (Gibco Company, USA) plus 1% pen/strep (Gibco Company, USA)) was added to terminate the digestion. After filtration through a 200-mesh sieve, the mixture was centrifuged at 300 × *g* for 10 min, and the resulting precipitate was used as the SVF of adipose tissue (stromal vascular fraction, which is readily obtained from human lipoaspirate samples using enzymatic digestion) [5].

The SVF was resuspended in complete medium, inoculated into a cell culture flask as primary cells (P0), and cultured in a 5% CO_2_ incubator. The medium was changed every 3 days until the cells reached 80–90% confluence, at which time they were passaged at a ratio of 1 : 3. P3 generation cells were characterized by flow cytometry and subjected to adipogenic and osteogenic induction [6].

### 2.3. Extraction and Culture of ADSC-Derived Exosomes

P3 passage ADSCs were used for culture. After the cells reached 70–80% confluence, they were washed twice with PBS, and the culture medium was replaced with serum-free medium. After 48 h, the supernatant was collected and processed and the obtained exosome solution was stored at -80°C. Next, the suspended cells were removed by centrifugation (300 × *g*, 10 min, 4°C). Cell debris was removed by further centrifugation (10,000 × *g*, 60 min, 4°C). The cell culture supernatant was filtered through a 0.22 *μ*m filter. The filtered cell supernatant was placed in a centrifuge bottle, weighed, and centrifuged at 120,000 × *g* and 4°C for 2 h (Optima XPN-100 Beckman Coulter, Brea, CA, USA). PBS was added to the centrifuge bottle to wash the exosome solution obtained by ultracentrifugation. The exosome solution was washed in the centrifuge bottle again and weighed, and the ultracentrifugation step was repeated. The supernatant was carefully removed, and Dulbecco's PBS was gently added to resuspend the exosome pellet, yielding the exosome solution. This solution was aliquoted into Eppendorf tubes and stored at -80°C until use [7].

### 2.4. Characterization of ADSC-Derived Exosomes

Currently, there is no standardized process for the isolation and characterization of exosomes. Most researchers have used a combination of two or more methods for characterization, such as flow cytometric measurement of surface markers, electron microscopy, and NTA (Nanoparticle Tracking Analysis). The exosome suspensions were characterized by transmission electron microscopic analysis, flow cytometric analysis of CD63 and CD81, and NanoSight particle electron microscopy (Malvern Analytical, Malvern, UK).

The size distribution and concentration of ADSC-derived exosomes were analyzed by NTA using NanoSight NS300 (Malvern Panalytical, Malvern, UK). The solution of ADSC-derived exosomes was injected into the laser chamber with a 1 ml syringe and recorded three times for 30 s. The mode, average size, and concentration of ADSC-derived exosomes were determined by using the NTA 3.0 software.

Morphology of ADSC-derived exosomes was visualized by transmission electron microscopy (TEM) using a JEM-1200EX electron microscope (JEOL, Tokyo, Japan). First, ADSC-derived exosomes were absorbed in a formvar/carbon-coated grid for 10 min and fixed with 2% paraformaldehyde. After negative staining with 2% uranyl acetate for 10 min, ADSC-derived exosomes were observed by TEM operated at 60 kV. Zeta potential was measured using a Zetasizer Nano ZS (Malvern Panalytical, Malvern, UK).

The nonstained exosome was used as a negative control and labeled NC. CD63 and CD81 antibodies (Becton, Dickinson and Company, USA) were stained and labeled as CD63 and CD81, respectively. CD63 and CD81 antibodies were detected by flow cytometry (BD Accuri C6 Flow Cytometer, Becton, Dickinson and Company, USA).

### 2.5. Coculture of ADSC-Derived Exosomes with Human Fibroblasts

Protein concentration of the exosome suspensions was determined by BCA assay (Beijing ComWin Biotech Co., Beijing, China). Concentrations were divided into 12.5, 25, 50, 100, and 200 *μ*g/mL for coculture with human fibroblasts. The CCK8 assay was used to measure the rate of cell proliferation at 1, 3, 5, and 7 days, and the optimal exosome concentration was screened.

### 2.6. Animal Experiments

SD rats (15 female rats, aged 6 weeks, weighing 100 ± 5 g, provided by Guangdong Medical Laboratory Animal Center, approval number SYXK (Guangzhou, China) 2017-0174) were used in the study. They were raised in the Experimental Animal Management Center of Jinan University in a specific pathogen-free environment.

### 2.7. Animal Groups and Construction of a Skin Photoaging Model in SD Rats

Fifteen 6-week-old SD rats were housed in the Experimental Animal Management Center of Jinan University. They were conditioned for 1 week in a specific pathogen-free environment and randomly divided into a normal control group, model group, normal saline control group, and ADSC-derived exosome treatment group. Each group contains 3 SD rats. Except for the normal control group, experimental groups were irradiated with a customized UVB light box (UVB light wavelength 290–320 nm, 15 W, 2 bulbs, light box height 50 cm); SD rats were free to move in the light box. The irradiation protocol was as follows [8]: 60 mJ/cm^2^ in week 1, 120 mJ/cm^2^ in week 2, 180 mJ/cm^2^ in week 3, and 240 mJ/cm^2^ in weeks 4–8, 5 times per week for 8 weeks, with a total irradiation dose of 7.8 J/cm^2^.

### 2.8. ADSC-Derived Exosome Injection Treatment

UVB irradiation was terminated 3 days before injection. According to the injection method and dose described above, SD rats were anesthetized with 10% chloral hydrate, and 100 *μ*L of exosome suspension at different concentrations was injected subcutaneously into the dorsal skin of the rats. The normal saline control group was injected with an equivalent dose of normal saline. A piece of tissue 6 mm in diameter was punched using a skin biopsy punch at 7, 14, and 28 days after injection, and the sample was fixed in 4% paraformaldehyde for paraffin sectioning. All animals were sacrificed after 28 days (rats were euthanized), and the remaining skin tissue was frozen in a liquid nitrogen tank until use.

### 2.9. Skin Sample Preparation and Measurement

Skin samples were fixed in 4% paraformaldehyde for 48 h, dehydrated and cleared, and embedded in paraffin. Paraffin blocks were serially sectioned (4 *μ*m) and adhered to slides treated with polylysine. Each group of paraffin sections was deparaffinized with xylene, hydrated through an ethanol gradient, stained with hematoxylin and 0.5% eosin solution, dehydrated through an ethanol gradient, and sealed with neutral gum. Slides were photographed using an inverted phase-contrast microscope (Olympus Corporation, Japan). The thickness of the epidermis and dermis was measured using ImageJ software (NIH, Bethesda, MD, USA).

### 2.10. Western Blotting

Tissues were homogenized in RIPA buffer containing a protease inhibitor and then sonicated for 15 minutes at 4°C to extract proteins, followed by protein concentration detection. Proteins were separated by 10% SDS-polyacrylamide gel electrophoresis (PAGE) and then transferred onto nitrocellulose membranes. After blocking, membranes were incubated with primary antibodies of collagen I (1 : 1000; Abcam), collagen III (1 : 1000), MMP-1 (1 : 1000), and MMP-3 (1 : 1000) overnight at 4°C and then with an HRP-linked secondary antibody. Detection was performed using ECL Prime (GE Healthcare, Little Chalfont, UK). GAPDH were used as the loading control.

### 2.11. Real-Time PCR

The relative expression levels of type I collagen, type III collagen, MMP-1, and MMP-3 mRNA were determined. Skin tissue (50 mg) stored in liquid nitrogen was collected. Total RNA was extracted from the skin tissue sample. The quality of extracted total RNA was determined by 1% gel electrophoresis. After determining the concentration, reverse transcription was performed using a reverse transcription kit (Toyobo, Osaka, Japan). After reverse transcription, PCR detection of cDNA was performed using the SYBR Green I fluorescent chimeric dye method. Primer sequences were as follows: internal reference *β*-actin upstream primer: 5′-GAGTACAACCTTCTTGCAGCTC-3′, downstream primer: 5′-CATACCCACCATCACACCCTG-3′, and amplicon length: 198 base pairs (bp); type I collagen upstream primer: 5′-GATGGACTCAACGGTCTCCC-3′, downstream primer: 5′-CGGCCACCATCTTGAGACTT-3′, and amplicon length: 143 bp; type III collagen upstream primer: 5′-CTGAAGGGCAGGGAACAACT-3′, downstream primer: 5′-ATCCCGAGTCGCAGACACATA-3′, and amplicon length: 270 bp; MMP-1 upstream primer: 5′-GGCTACCAGCTCATACAGTTTCC-3′, downstream primer: 5′-CCTCATAGCACTCAGGGTTTCAG-3′, and amplicon length: 223 bp; and MMP-3 upstream primer: 5′-CAGGCATTGGCACAAAGGTG-3′, downstream primer: 5′-CTGAAACACACGACGCCTTC-3′, and amplicon length: 174 bp. The 2^-*ΔΔ*Ct^ method was used for calculations.

### 2.12. Immunohistochemical Analysis

Paraffin sections of skin samples were collected, deparaffinated with xylene, hydrated through an ethanol gradient, placed in sodium citrate buffer (pH 6.0), and transferred to a microwave oven for antigen retrieval for 10 min. After natural cooling to room temperature, slides were incubated in 0.3% Triton X-100 for 15 min at room temperature. The slides were immersed in 3% H_2_O_2_ for 20 min to remove endogenous catalase, followed by blocking with goat serum blocking solution for 20 min at room temperature. Rabbit anti-rat Ki67 polyclonal primary antibody working solution (1 : 100 dilution, Abcam, Cambridge, UK) was added to the slides, which were incubated at 4°C overnight. On the next day, the slides were washed with PBS, a goat anti-rabbit IgG antibody (Wuhan Servicebio Technology Co., Ltd., Wuhan, China) was added, and the slides were incubated at 37°C for 30 min. A DAB color reagent was used for color development. Hematoxylin counterstaining, dehydration through an ethanol gradient, and xylene clearing were performed. The slides were sealed with neutral balsam and observed by microscopy. Brown-yellow coloration in the nucleus was considered positive staining. Ten high-magnification (×100) fields were randomly selected from each slide, and the number of positive cells in each field was determined.

### 2.13. Statistics

SPSS 13.0 statistical software (SPSS, Inc., Chicago, IL, USA) was used for analysis. Measurement data are expressed as the mean ± standard deviation (mean ± SD). The data from each group were analyzed by analysis of variance in a completely randomized design. Differences with *p* < 0.05 were considered statistically significant.

## 3. Results

### 3.1. Morphological Observation and Characterization of ADSCs Cultured *In Vitro*

**Figure 1:**
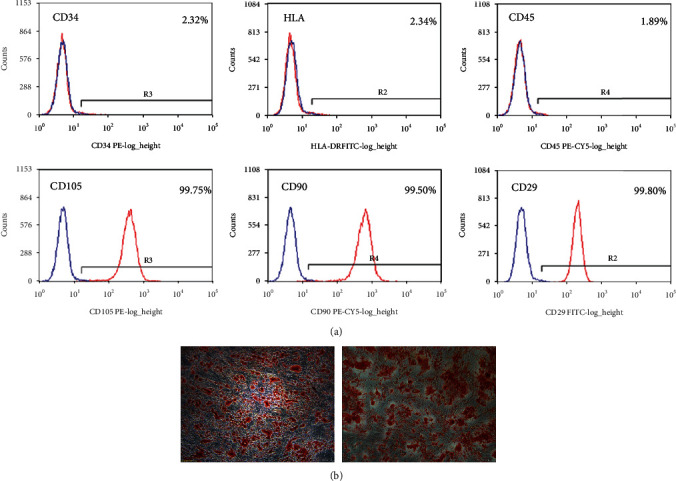
Morphological observation and characterization of ADSCs cultured *in vitro*. (a) Flow cytometry labeling of surface markers showed positive expression of CD29, CD90, and CD105 with positive rates of 99.98%, 99.5%, and 99.75%, respectively. CD34, CD45, and human leukocyte antigen-DR expression was negative with expression rates of 2.32%, 1.89%, and 2.34%, respectively. (b) After adipogenic induction, P3 cells were stained with Oil Red O, and oil droplet-like adipose tissue was observed by microscopy. After osteogenic induction, Alizarin Red staining showed that aggregated bone tissue was stained red in microscopy analysis.

Primary cultured ADSCs were fully extended after adherence, exhibited a long, fusiform morphology, and grew individually or in colonies. Flow cytometry labeling of surface markers showed positive expression of CD29, CD90, and CD105 with positive rates of 99.98%, 99.5%, and 99.75%, respectively. CD34, CD45, and human leukocyte antigen-DR expression was negative with expression rates of 2.32%, 1.89%, and 2.34%, respectively (Figure 1(a)). After adipogenic induction, P3 cells were stained with Oil Red O, and oil droplet-like adipose tissue was observed by microscopy. After osteogenic induction, Alizarin Red staining showed that aggregated bone tissue was stained red, demonstrating that the cells obtained in this experimental group were ADSCs (Figure 1(b)).

### 3.2. Characterization of ADSC-Derived Exosomes

**Figure 2:**
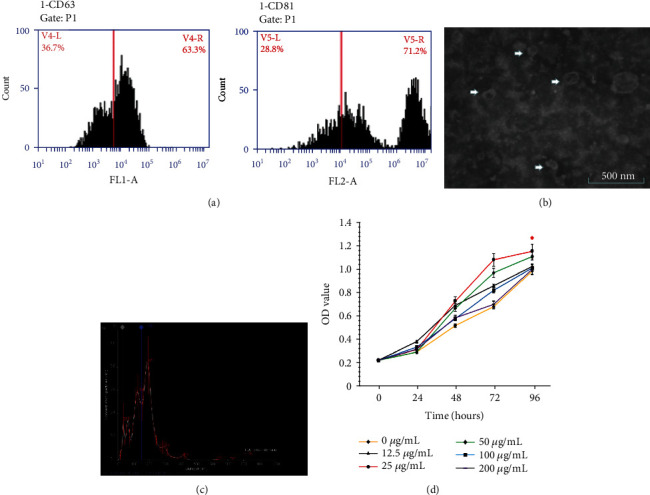
Characterization of ADSC-derived exosomes. (a) The results of flow cytometric measurement of surface markers showed positive expression of CD63 and CD81 in the ultracentrifugation extract. (b) Transmission electron microscopy showed that the precipitate obtained by ultracentrifugation contained biconcave disc-shaped vesicles with diameters of 30–150 nm. (c) The exosome particle size distribution and particle concentration measured using NanoSight. Concentrations of ADSC-derived exosomes were divided into 12.5, 25, 50, 100, and 200 *μ*g/mL for coculture with fibroblasts. The CCK8 assay was used to measure the rate of cell proliferation at 1, 3, 5, and 7 days. (d) When the exosome protein concentration was 25 *μ*g/mL, the proliferation rate of the fibroblasts was increased significantly.

Currently, there is no standardized process for the isolation and characterization of exosomes. Most researchers have used a combination of two or more methods for characterization, such as flow cytometric measurement of surface markers, electron microscopy, and NTA. CD63 and CD81 belong to the exosome surface tetraspanin protein superfamily and are the most commonly used proteins for identifying exosomes. The results of flow cytometric measurement of surface markers showed positive expression of CD63 and CD81 in the ultracentrifugation extract (Figure 2(a)). Transmission electron microscopy showed that the precipitate obtained by ultracentrifugation contained biconcave disc-shaped vesicles with diameters of 30–150 nm, which is consistent with the morphological characteristics of exosomes (Figure 2(b)).  Figure 2(c) shows the exosome particle size distribution and particle concentration measured using NanoSight. Particles in the suspension showed a multimodal distribution with a particle size of 120.9 ± 42.9 nm. Most particles were in the range of exosomes (30–150 nm). The particles with the highest content had a diameter of approximately 114 nm; of these, 10.7% of particles were in the 30–150 nm range.

The results of surface marker flow cytometry showed that the precipitate obtained by ultracentrifugation was positive for exosome surface marker proteins CD63 and CD81. NanoSight analysis revealed that most microparticles had a diameter in the range of exosomes. Therefore, the precipitate obtained by ultracentrifugation contained exosomes released by ADSCs.

### 3.3. Proliferation Curves of Coculture of Fibroblasts with Different Concentrations of ADSC-Derived Exosomes

Concentrations of ADSC-derived exosomes were divided into 12.5, 25, 50, 100, and 200 *μ*g/mL for coculture with fibroblasts. The CCK8 assay was used to measure the rate of cell proliferation at 1, 3, 5, and 7 days.  Figure 2(d) shows that when the exosome protein concentration was 25 *μg*/mL, the proliferation rate of the fibroblasts was increased significantly. ANOVA for repeated measurement design was performed in two groups with 0 g/mL and 25 g/mL. The results showed that the difference between the two dose groups changed with time, and the difference was statistically significant (*F* = 64868.00, *p* < 0.01).

### 3.4. Epidermal Thickness in the Skin of SD Rats after Treatment with ADSC-Derived Exosomes

**Figure 3:**
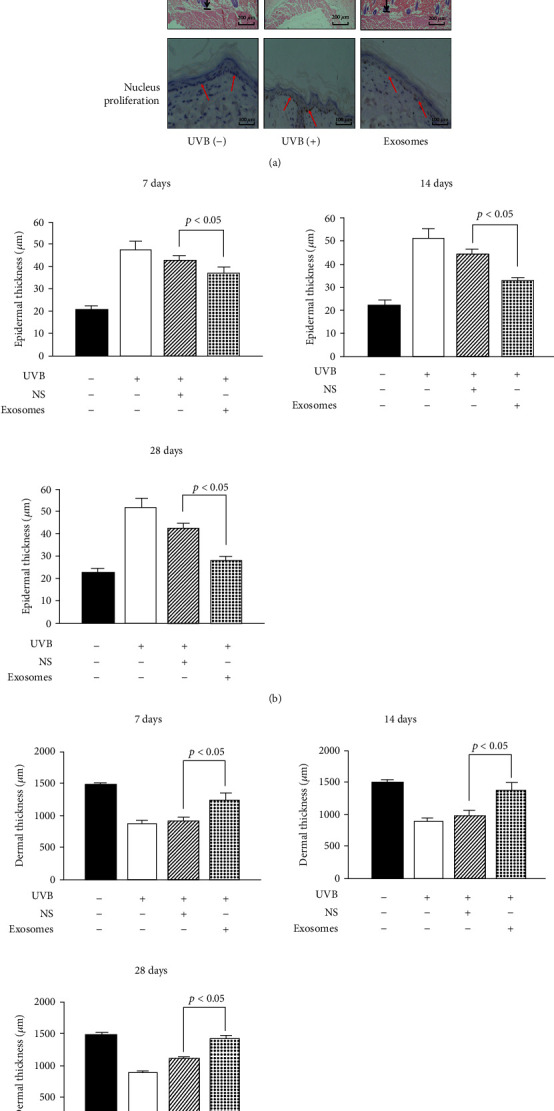
Skin changes of SD rats after treatment with ADSC-derived exosomes. (a) HE staining showed the changes in epidermal and dermal thickness of SD rats exposed to UVB 28 d after ADSC-derived exosome treatment (×100). Ki67 immunostaining showed the proliferation of basal cells of SD rats exposed to UVB and those treated with ADSC-derived exosomes for 28 days (×100). (b) The thickness of the epidermis in the ADSC-derived exosome treatment group was less than that in the control group at 7 days, 14 days, and 28 days after treatment (*p* < 0.05), indicating that the ADSC-derived exosome treatment reduced the epidermal layer thickness of photoaged skin. (c) The thickness of the dermis in the ADSC-derived exosome treatment group was higher than that in the control group at 7 days, 14 days, and 28 days after treatment (*p* < 0.05), indicating that the ADSC-derived exosome treatment increased the dermal layer thickness of photoaged skin.

Hematoxylin and eosin staining showed that the entire epidermal layer of the skin was significantly thickened after UVB irradiation and changed after treatment with ADSC-derived exosomes (Figure 3(a)). Statistical analysis showed that the thickness of the epidermis in the ADSC-derived exosome treatment group was less than that in the control group at 7, 14, and 28 days after treatment (*p* < 0.05), indicating that ADSC-derived exosome treatment reduced epidermal layer thickness of photoaged skin (Figure 3(b)).

### 3.5. Dermal Thickness in the Skin of SD Rats after Treatment with ADSC-Derived Exosomes

Hematoxylin and eosin staining showed that the entire dermal layer of the skin was significantly thinned after UVB irradiation and changed after treatment with ADSC-derived exosomes (Figure 3(a)). Statistical analysis showed that the thickness of the dermis in the ADSC-derived exosome treatment group was higher than that in the control group at 7, 14, and 28 days after treatment (*p* < 0.05), indicating that ADSC-derived exosome treatment increased the dermal layer thickness of photoaged skin (Figure 3(c)).

### 3.6. Analysis of Nucleus Proliferation in Stratum Basale Cells of the Epidermis

After UVB irradiation, the number of nuclei in a proliferating state in the stratum basale of the epidermis was significantly increased and the arrangement of these cells was disordered. After treatment with ADSC-derived exosomes, the number of nuclei in a proliferating state in the stratum basale was significantly reduced. After 7 days of treatment, this number in the ADSC-derived exosome treatment group was reduced, indicating that ADSC-derived exosome treatment can effectively inhibit abnormal proliferation of stratum basale cells caused by UVB irradiation (Figure 3(a)).

### 3.7. Changes in Relative Expression of Type I Collagen, Type III Collagen, MMP-1, and MMP-3 mRNA in the Dermis

**Figure 4:**
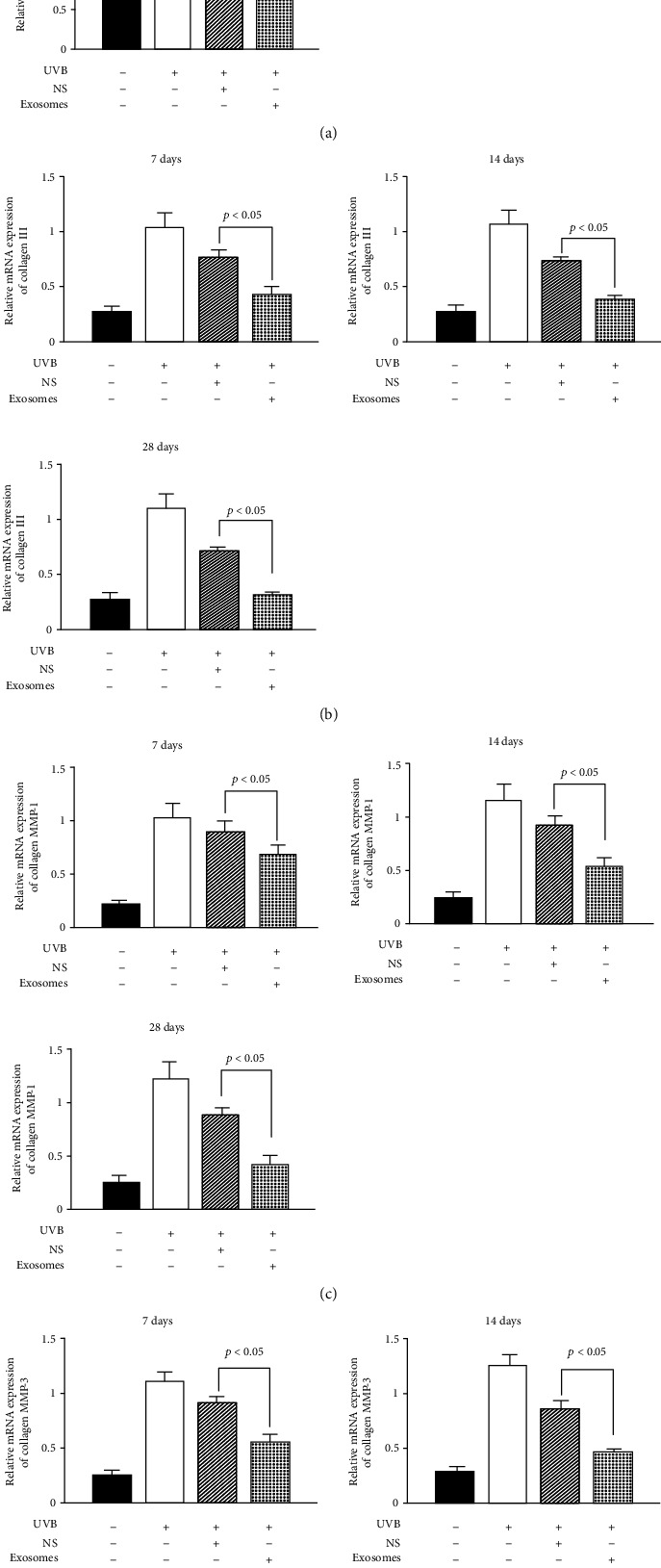
Changes in relative expression of type I collagen, type III collagen, MMP-1, and MMP-3 mRNA in the dermis. (a) Real-time PCR showed the relative mRNA expression of collagen I of SD rats exposed to UVB 28 d after ADSC-derived exosome treatment. Mean ± SD. *n* = 5. ^∗^*p* < 0.05 vs. UVB(-) group; ^△^*p* < 0.05 vs. UVB(+) group. (b) Real-time PCR showed the relative mRNA expression of collagen III of SD rats exposed to UVB 28 d after ADSC-derived exosome treatment. Mean ± SD. *n* = 5. ^∗^*p* < 0.05 vs. UVB(-) group; ^△^*p* < 0.05 vs. UVB(+) group. (c) Real-time PCR showed the relative mRNA expression of MMP-1 of SD rats exposed to UVB 28 d after ADSC-derived exosome treatment. Mean ± SD. *n* = 5. ^∗^*p* < 0.05 vs. UVB(-) group; ^△^*p* < 0.05 vs. UVB(+) group. (d) Real-time PCR showed the relative mRNA expression of MMP-3 of SD rats exposed to UVB 28 d after ADSC-derived exosome treatment. Mean ± SD. *n* = 5. ^∗^*p* < 0.05 vs. UVB(-) group; ^△^*p* < 0.05 vs. UVB(+) group.

After 28 days of treatment, the concentration of type I collagen in the dermis of the treatment group was increased, while type III collagen was decreased, and the difference in relative expression of mRNA was significant (*p* < 0.05). The relative expression of mRNA of MMP-1 and MMP-3, which are involved in collagen degradation, was decreased; only the exosome treatment group showed a significant difference (*p* < 0.05). This indicates that ADSC-derived exosomes can upregulate the relative expression of type I collagen mRNA and downregulate the expression of type III collagen, MMP-1, and MMP-3 mRNA (Figures 4(a)–Figure 4(d)).

### 3.8. Changes in Relative Protein Expression of Type I Collagen, Type III Collagen, MMP-1, and MMP-3 mRNA in the Dermis

**Figure 5:**
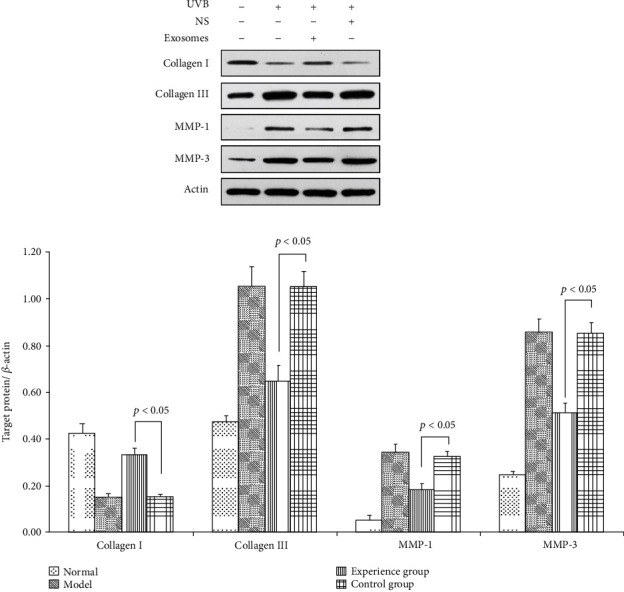
Changes in relative protein expression of type I collagen, type III collagen, MMP-1, and MMP-3 mRNA in the dermis. Western blotting showed the relative protein expression of collagen I of SD rats exposed to UVB 28 d after ADSC-derived exosome treatment. Mean ± SD. *n* = 5. ^∗^*p* < 0.05 vs. UVB(-) group; ^△^*p* < 0.05 vs. UVB(+) group. Western blotting showed the relative protein expression of collagen III of SD rats exposed to UVB 28 d after ADSC-derived exosome treatment. Mean ± SD. *n* = 5. ^∗^*p* < 0.05 vs. UVB(-) group; ^△^*p* < 0.05 vs. UVB(+) group. Western blotting showed the relative protein expression of MMP-1 of SD rats exposed to UVB 28 d after ADSC-derived exosome treatment. Mean ± SD. *n* = 5. ^∗^*p* < 0.05 vs. UVB(-) group; ^△^*p* < 0.05 vs. UVB(+) group. Western blotting showed the relative protein expression of MMP-3 of SD rats exposed to UVB 28 d after ADSC-derived exosome treatment. Mean ± SD. *n* = 5. ^∗^*p* < 0.05 vs. UVB(-) group; ^△^*p* < 0.05 vs. UVB(+) group.

After 28 days of treatment, the concentration of type I collagen in the dermis of the treatment group was increased, while type III collagen was decreased, and the difference in relative expression of protein was significant (*p* < 0.05). The relative expression of protein of MMP-1 and MMP-3, which are involved in collagen degradation, was decreased; only the exosome treatment group showed a significant difference (*p* < 0.05). This indicates that ADSC-derived exosomes can upregulate the relative expression of type I collagen protein and downregulate the expression of type III collagen, MMP-1, and MMP-3 protein (Figure 5).

## 4. Discussion

We demonstrated that ADSC-derived exosomes can effectively improve thickening of the epidermis and stratum corneum of the epidermis, abnormal proliferation of stratum basale cell nuclei, thinning of the dermis, and other phenomena caused by photoaging and thus may be useful for counteracting skin photoaging. Normal renewal of the epidermis occurs in approximately 28 days. However, in the event of foreign body invasion or destruction of the stratum corneum, cell division in the stratum basale is accelerated, thus accelerating the rate of renewal, eliminating foreign bodies, and facilitating repair. Chemical or physical stimulation of the skin can lead to epidermal hyperplasia and abnormal keratinization, resulting in abnormal thickening of the stratum corneum. In our study, the epidermal tissue of the SD rats was significantly thickened after UVB irradiation. This phenomenon occurred simultaneously in the stratum basale and stratum corneum of the epidermis, with the proportion of stratum corneum cells showing a significant increase. Additionally, the arrangement of cells in the stratum basale of the epidermis was disordered after UVB irradiation, and some cells exhibited changes in nuclear morphology. UV-induced DNA damage of keratinocytes can cause abnormal cell proliferation in the absence of repair [9, 10]. After ADSC-derived exosome collection and subcutaneous injection, the number of cells in a proliferative state in the stratum basale of the epidermis was significantly reduced. This effect was evident in the ADSC-derived exosome treatment group, and the number of nuclei in a proliferative state was generally the same as that in normal skin tissue, indicating that ADSC-derived exosomes have important regulatory effects in counteracting UV-induced damage to the epidermis; however, the mechanism of action requires further investigation. These effects may counteract the UV-induced epidermal damage and improve the thickening of epidermal tissue through two main pathways. The first occurs through an effective inhibition of the abnormal proliferation of stratum basale cells, which in turn effectively inhibits the hyperkeratosis of epidermal cells. The second involves a regulatory recalibration that boosts the keratinization and renewal rates of epidermal tissue cells, thereby promoting the renewal of epidermal cells and restoring epidermal homeostasis. Both mechanisms ameliorate the effects of skin photoaging and promote wound healing, and photoaging and wound healing are physiological processes of the skin that share certain mechanisms. Senescence-associated *β*-galactosidase (SA-*β*-Gal) activity was assessed as a marker of natural senescence. Exosomal therapy is closely related to *β*-galactosidase which has been reported to reduce the expression level of SA-*β*-Gal [11]. It has been reported that ADSC-derived exosomes promote fibroblast proliferation, angiogenesis, and growth and epithelialization of granulation tissue, thereby promoting wound healing [12, 13]. This experiment uses human ADSC-EXO, because the exosomes are characterized by low immunogenicity and have an application potential due to long-term preservation, stable physiological properties, wide range of sources, and large-scale extraction.

In our experiments, subcutaneous injection of ADSC-derived exosomes increased dermal thickness in SD rats, promoted the expression of type I collagen mRNA in the dermis, and inhibited the expression of type III collagen mRNA. Collagen, primarily type I (85–95%) and type III collagen (10–15%) accounts for approximately 90% of the protein content in the human dermis. With increasing age, type I collagen content in the skin decreases, while the proportion of type III collagen increases; this is more pronounced in sites exposed to UVB. The decreased collagen content and change in the collagen ratio of the different components cause the skin to become lax and collapse, resulting in wrinkle formation [14]. ADSC-derived exosomes can effectively improve the proportion of type I/III collagen in the dermal tissue, causing it to resemble young, healthy skin tissue. ADSC-derived exosomes can repair dermal tissue by promoting collagen secretion by fibroblasts [15, 16]. MMPs are a family of zinc-containing proteases that cause tissue damage by degrading the extracellular matrix, thereby leading to wrinkle formation [17]. Excessive UV irradiation can increase MMP secretion by keratinocytes, fibroblasts, and inflammatory cells, among others [18]. The secretion of MMP-1, MMP-3, and MMP-9 can be induced by UV irradiation. With respect to type I collagen, which is closely related to skin quality, MMP-1 initiates the breakdown of collagen fibers and MMP-3 further degrades collagen. The MMP-3 content in dermal tissue directly affects the degradation efficiency of collagen. We observed that subcutaneous injection of ADSC-derived exosomes effectively reduced the relative expression of MMP-3 mRNA in the dermal tissue, which may be because of the reduced rate of collagen degradation and corrected imbalance between collagen synthesis and degradation, thereby increasing collagen content and improving skin aging.

Skin repair and regeneration require synergy between various tissues and cells to replace, repair, and reconstruct defects in cell structures and tissue layers. Exosomes are important factors affecting multiple processes and cells to promote skin repair and regeneration, such as promoting fibroblast proliferation and migration, promoting angiogenesis, regulating local inflammatory response to injury, promoting collagen synthesis in the early stages of wound healing to improve the speed of repair, and inhibiting collagen synthesis in late stages to inhibit scar tissue formation.

In recent years, an increasing number of studies have focused on skin photoaging research and treatment, including investigations of the application of stem cells. Other studies confirmed that ADSCs can promote fibroblast proliferation, secretion, and migration through paracrine and autocrine effects. Additionally, stem cells can promote angiogenesis to participate in the repair and structural reconstruction of damaged skin tissue. Previous studies also suggested that ADSCs can promote collagen secretion and other pathways to improve skin aging through antioxidative effects and fibroblast activation. Exosomes are structures in cells that are important for regulating stem cell secretion and paracrine secretion. Numerous studies have recently been conducted on vascular remodeling. Studies have also shown that exosomes can induce macrophage polarization and promote wound repair. This occurs through microRNAs contained in stem cell-derived exosomes, which can regulate cell proliferation and differentiation and promote wound healing. Little is known about the effect of exosomes on photoaging skin. We, therefore, used UVB to induce skin photoaging in an SD rat model followed by subcutaneous injection of exosomes for skin photoaging treatment to observe the effect of exosomes on photoaging skin.

## 5. Conclusions

In summary, our study confirmed the potential of ADSC-derived exosomes for improving skin photoaging from epigenetic, histological, and mRNA expression perspectives based on changes in dermal and epidermal thickness and structure, relative mRNA expression of dermal collagens, MMP-1 and MMP-3, and the proliferative capacity of nuclei in stratum basale cells, providing a theoretical basis for the clinical application of ADSC-derived exosomes for treating skin photoaging.

## Data Availability

The research article data used to support the findings of this study are included within the article.

## Supplementary Materials

Supplementary Materials Statistical analysis showed that in the ADSC-derived exosome treatment group, the number of nuclei in the proliferated basal layer was less than that in the control group 28 days after treatment (*p* < 0.05). It indicates that ADSC-derived exosome treatment reduces the number of nuclei in the base layer of the proliferative state.
