# Supplementary figures and images for: Antiaging Properties of Exosomes from Adipose-Derived Mesenchymal Stem Cells in Photoaged Rat Skin
Source: Biomed Res Int. 2020 Dec 21;2020:6406395. doi: 10.1155/2020/6406395 (PMC7769639; doi:10.1155/2020/6406395)

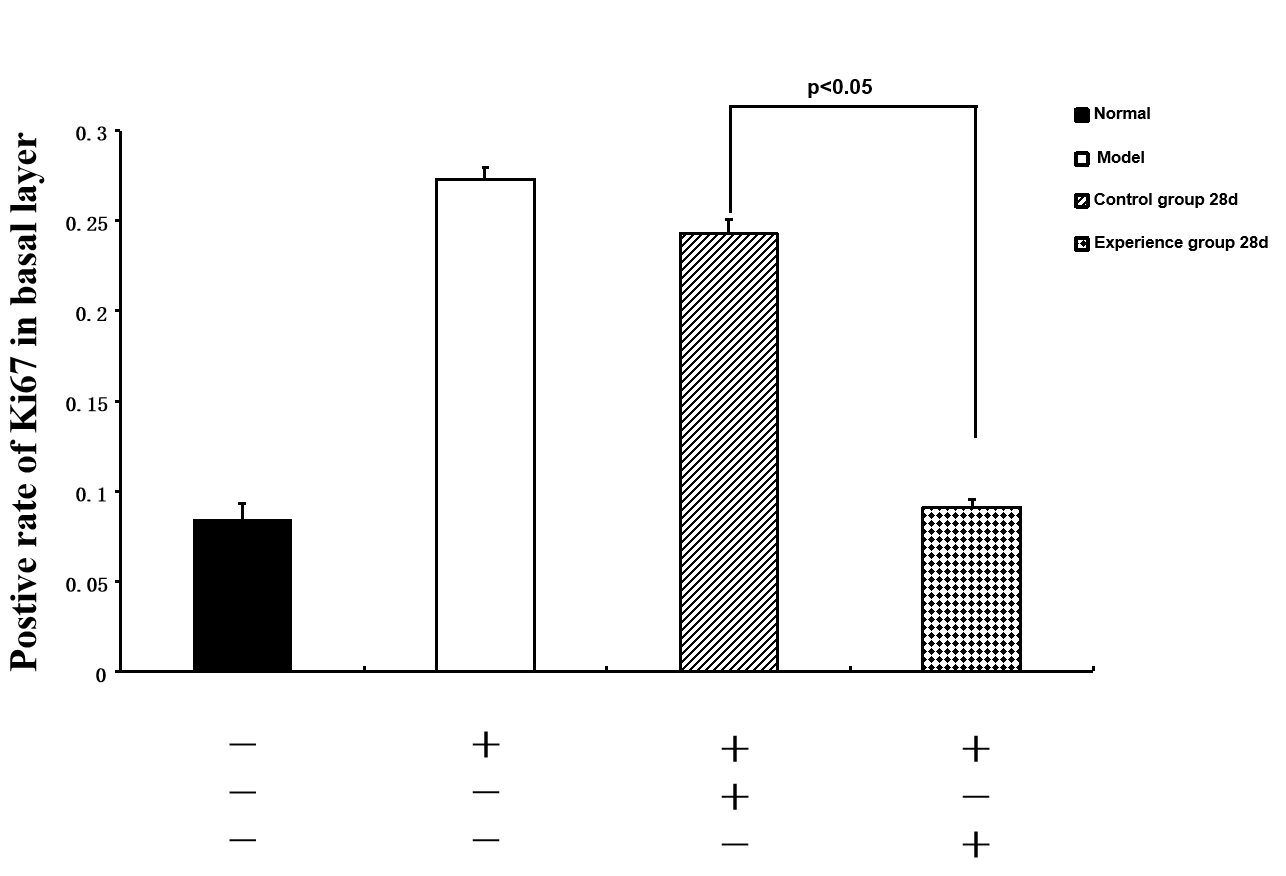

Supplement: Supplementary Materials — Statistical analysis showed that in the ADSC-derived exosome treatment group, the number of nuclei in the proliferated basal layer was less than that in the control group 28 days after treatment (p < 0.05). It indicates that ADSC-derived exosome treatment reduces the number of nuclei in the base layer of the proliferative state. [file 6406395.f1.tif]
